# EPR Investigations to Study the Impact of Mito-Metformin on the Mitochondrial Function of Prostate Cancer Cells

**DOI:** 10.3390/molecules27185872

**Published:** 2022-09-10

**Authors:** Donatienne d’Hose, Barbara Mathieu, Lionel Mignion, Micael Hardy, Olivier Ouari, Bénédicte F. Jordan, Pierre Sonveaux, Bernard Gallez

**Affiliations:** 1 Biomedical Magnetic Resonance, Louvain Drug Research Institute (LDRI), Université Catholique de Louvain (UCLouvain), 1200 Brussels, Belgium; 2 Institut de Chimie Radicalaire UMR 7273, Aix-Marseille Université/CNRS, 13013 Marseille, France; 3 Pole of Pharmacology and Therapeutics, Institut de Recherches Expérimentales et Cliniques (IREC), Université Catholique de Louvain (UCLouvain), 1200 Brussels, Belgium; 4 Walloon Excellence in Life Sciences and Biotechnology (WELBIO) Research Institute, 1300 Wavre, Belgium

**Keywords:** EPR, ESR, tumor oxygenation, tumor hypoxia, oxygen consumption, mitochondrial ROS, oximetry, cancer, irradiation

## Abstract

Background: Mito-metformin10 (MM10), synthesized by attaching a triphenylphosphonium cationic moiety via a 10-carbon aliphatic side chain to metformin, is a mitochondria-targeted analog of metformin that was recently demonstrated to alter mitochondrial function and proliferation in pancreatic ductal adenocarcinoma. Here, we hypothesized that this compound may decrease the oxygen consumption rate (OCR) in prostate cancer cells, increase the level of mitochondrial ROS, alleviate tumor hypoxia, and radiosensitize tumors. Methods: OCR and mitochondrial superoxide production were assessed by EPR (9 GHz) in vitro in PC-3 and DU-145 prostate cancer cells. Reduced and oxidized glutathione were assessed before and after MM10 exposure. Tumor oxygenation was measured in vivo using 1 GHz EPR oximetry in PC-3 tumor model. Tumors were irradiated at the time of maximal reoxygenation. Results: 24-hours exposure to MM10 significantly decreased the OCR of PC-3 and DU-145 cancer cells. An increase in mitochondrial superoxide levels was observed in PC-3 but not in DU-145 cancer cells, an observation consistent with the differences observed in glutathione levels in both cancer cell lines. In vivo, the tumor oxygenation significantly increased in the PC-3 model (daily injection of 2 mg/kg MM10) 48 and 72 h after initiation of the treatment. Despite the significant effect on tumor hypoxia, MM10 combined to irradiation did not increase the tumor growth delay compared to the irradiation alone. Conclusions: MM10 altered the OCR in prostate cancer cells. The effect of MM10 on the superoxide level was dependent on the antioxidant capacity of cell line. In vivo, MM10 alleviated tumor hypoxia, yet without consequence in terms of response to irradiation.

## 1. Introduction

**Figure 1:**
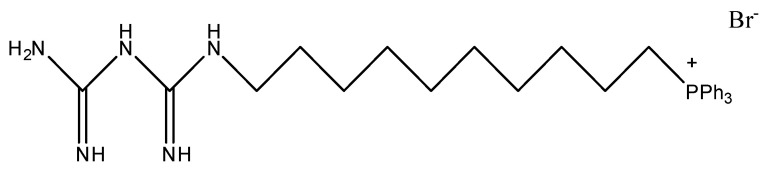
Chemical structure of Mito-metformin10 (MM10).

Mitochondria play a key role in cancer development. Many mitochondrial pathways, including oxidative phosphorylation, fatty acid, and glutamine metabolism, are altered in tumor cells, due to mutations in oncogenes and tumor suppressor genes. This results in metabolic reprogramming that sustains rapid cell proliferation [1,2]. The inhibition of mitochondrial function could potentially inhibit the proliferation of cancer cells and induce cytotoxicity [3]. In this context, metformin, the most prescribed antidiabetic drug, has received particular attention. At the origin, metformin was repurposed as an antitumor agent due to studies that discovered an association between decreased incidence of pancreatic cancer and metformin use in diabetic individuals [4,5,6]. It has been assumed that mitochondria are the principal target of metformin. The effects of metformin on cancer progression were attributed to its ability to inhibit mitochondrial complex I leading to inhibition of mitochondrial respiration and bioenergetic reprogramming [7,8,9,10,11]. Although antitumor activity was reported in rodents, concentrations of drugs required to achieve an antineoplastic activity of metformin in humans would require much higher concentrations of the drug in the serum than those obtained when treating patients for diabetes [4]. This observation led to the development of mitochondria-targeted metformin analogues where metformin has been anchored to the lipophilic cation triphenylphosphonium (TPP^+^) [6,11,12,13]. A series of metformin analogues was synthesized, and it was found that Mito-metformin10 (MM10, Figure 1), obtained by attaching TPP^+^ to metformin via a 10-carbon aliphatic side chain, was about 1000 times more efficacious than metformin at inhibiting cell proliferation in pancreatic ductal adenocarcinoma (PDAC) [12]. Using the Seahorse technology on pancreatic tumor cells, it was found that MM10 inhibited mitochondrial respiration [12]. In addition, the blockade of the electron transport chain (ETC) led an increase in superoxide production as revealed by fluorescence assays coupled to HPLC [12]. Finally, these authors also found that MM10 enhanced the radiosensitivity of pancreatic cancer cells in vitro [12].

In the present study, we focused on the mitochondrial function of prostate cancer cells and the possible alleviation of tumor hypoxia to render tumors more sensitive to irradiation in vivo. The rationale relies on the observation that the presence of hypoxia is a poor prognostic factor in prostate cancer and promotes radioresistance [14,15,16,17]. Tumor hypoxia may theoretically be alleviated by increasing the oxygen delivery or by decreasing the oxygen consumption rate (OCR) of cancer cells. A mathematical modelling suggested that strategies designed to decrease OCR would be more efficient than the manipulation of the oxygen supply [18]. Experimentally, several strategies designed to decrease the OCR of cancer cells succeeded in increasing the response to radiation therapy in tumor models, as reviewed in [19]. In this context, it has been described that metformin decreased the OCR and alleviate tumor hypoxia in HCT116 colorectal carcinoma, in POP-092S colon adenocarcinoma [20] and in A549 in non-squamous cell lung carcinoma [21]. In the Colo205 colorectal cancer model, metformin decreased hypoxia but had no additive effect on radiotherapy efficacy [22]. Because MM10 was previously found more efficient than metformin in the PDAC model, we made the hypothesis that the use of MM10 could be efficient in reprogramming tumor cell metabolism, alleviate tumor hypoxia and sensitize tumor cells to irradiation in prostate cancer where hypoxia is known as a major factor of resistance to radiation therapy. To study the effect of MM10 on the mitochondrial function and the tumor hypoxia, we used electron paramagnetic resonance (EPR) spectroscopy both in vitro and in vivo. The OCR was measured in vitro by measuring continuously the variation of the EPR linewidth of an oxygen sensitive soluble probe introduced in a suspension of prostate cancer cells in a sealed capillary tube [23,24]. Compared to other methods, the assessment of OCR by EPR was found more sensitive and reproducible than electrode-based or fluorimetric methods [24]. Compared to Seahorse XF technology that is performed on adherent cells, the OCR assessment by EPR method has the disadvantage of requiring cell detachment by trypsin or collagenase that may slightly alter the oxygen consumption (except if cells are loaded on microbeads) [25]. We also measured the mitochondrial superoxide production in vitro by using Mito-TEMPO-H, a cyclic hydroxylamine able to detect reactive oxygen species (ROS) in complex biological media [26,27,28]. In vivo, low frequency (1 GHz) EPR spectroscopy was used to measure oxygen directly in tumor models as this method has the unique capability to measure subtle variations in tissue oxygenation from the same site over long periods of time [29,30,31]. The radiosensitivity of prostate tumors was then checked by applying the irradiation at the timing of a maximal increase in tumor oxygenation induced by MM10 as identified by EPR oximetry.

## 2. Results

### 2.1. Ocr Is Decreased by MM10 in Prostate Cancer Cells

**Figure 2:**
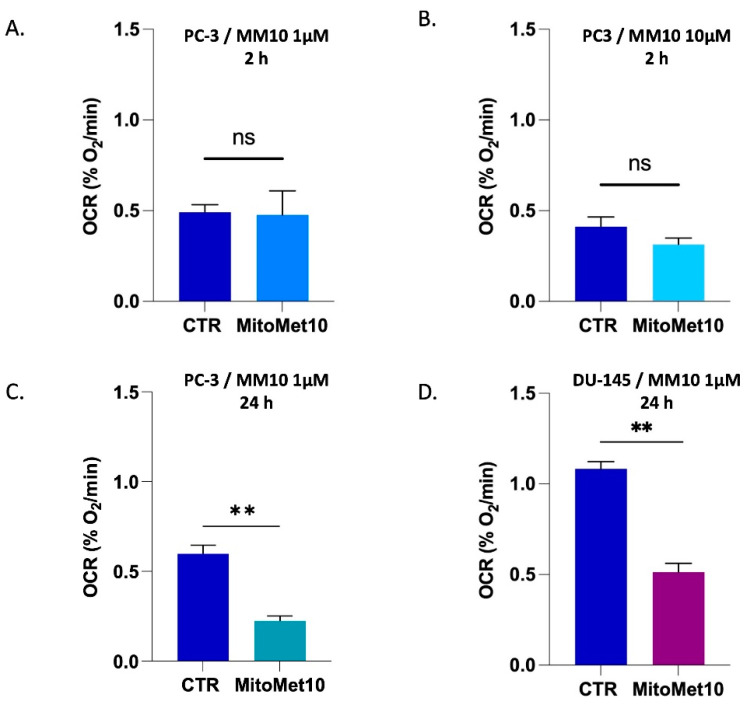
Impact of MM10 on the oxygen consumption rate (OCR) of prostate cancer cells measured by EPR respirometry. (**A**) Effect on OCR of PC-3 cells after 2 h exposure to MM10 (1 µM). (**B**) Effect on OCR of PC-3 cells after 2 h exposure to MM10 (10 µM). (**C**) Effect on OCR of PC-3 cells after 24 h exposure to MM10 (1 µM). (**D**) Effect on OCR of DU-145 cells after 24 h exposure to MM10 (1 µM). Bars represent means ± SEM (% O_2_/min), (**) *p* < 0.01, (ns) non-significant ,Student’s *t* test, N = 3.

The OCR was measured in two prostatic cancer cells lines, PC-3 and DU-145. To assess the OCR in the cancer cells, we performed in vitro EPR respirometry. The coefficient of variation of the OCR measurements carried out in this study was 8.6%. We first tested concentrations of 1 µM and 10 µM of MM10 for 2 h in PC-3 cells. No change in OCR was observed after this short time exposure (Figure 2A,B). We also measured the OCR when PC-3 cells were exposed to MM10 at 1 µM for 24 h and found out that OCR was significatively decreased (Figure 2C). These measurements were also repeated on a different prostatic cancer cell line, DU-145 cells. The basal OCR for untreated control DU-145 cells was larger than for PC-3 cells, consistent with a higher oxidative metabolism for DU-145 cells. For the same concentration and timing (1 µM MM10 for 24 h), the decrease in OCR was observed in DU145 cells (Figure 2D).

### 2.2. Mitochondrial Superoxide Production Is Increased after MM10 24 H Exposure in PC-3 but Not in DU-145 Cells

**Figure 3:**
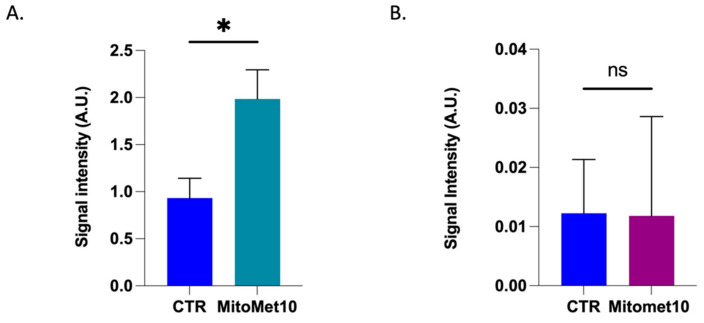
Impact of MM10 on mitochondrial superoxide level in prostate cancer cells measured by EPR spectroscopy. Mitochondrial superoxide was measured using mitoTEMPO-H as EPR sensor. Superoxide contribution to the signal was measured by making the difference between the signal intensities recorded in the absence and in the presence of PEGSOD2. (**A**) Impact on mitochondrial superoxide production of PC3-cells after 24 h exposure to MM10 (1 µM). (**B**) Impact on mitochondrial superoxide production of DU-145 cells after 24 h exposure to MM10 (1 µM). Bars represent means ± SEM, (*) *p* < 0.05, (ns) non-significant, Student’s *t* test, N = 3.

Because an increased superoxide production may be the result of a dysfunction in the mitochondrial electron transport chain (ETC) [32,33], we investigated the mitochondrial superoxide production by in vitro EPR spectroscopy. The assay is based on the oxidation of a hydroxylamine into a nitroxide. As several factors may contribute to the oxidation of the probe, we used PEG-SOD to measure the contribution of superoxide to the formation of the nitroxide. Previous studies have indeed shown that the pre-incubation of cells in the presence of PEG-SOD allows cellular uptake of the enzyme and intracellular scavenging of superoxide [34]. When exposed to MM10 at the concentration of 1 µM for 24 h, the level of mitochondrial superoxide was significatively increased in PC-3 cells (Figure 3A) but remained unchanged in DU-145 cells (Figure 3B). We also noticed that the basal mitochondrial superoxide production was much lower in DU-145 than in PC-3 cells. 

### 2.3. DU-145 Cells Have Higher Glutathione Levels and Are More Resistant to Oxidative Stress Than PC-3 Cells

**Figure 4:**
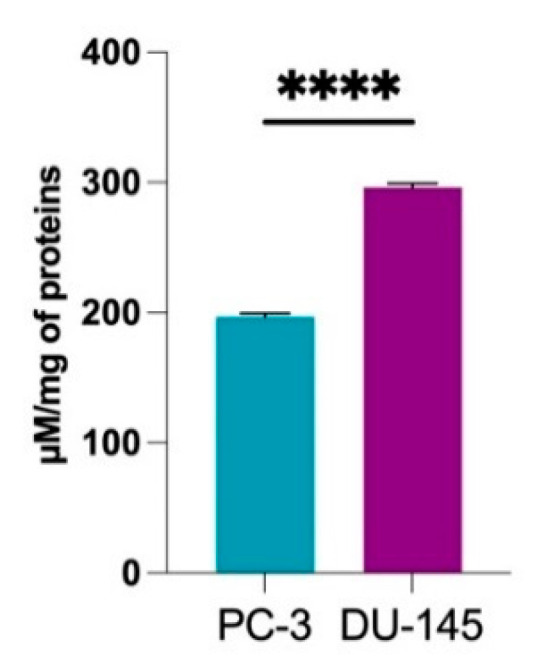
Basal level of total glutathione (reduced + oxidized) in different prostate cancer cell lines (PC-3 and DU-145). Bars represent means ± SEM (µM/mg of proteins), (****) *p* < 0.0001, Student’s *t* test, N = 3.

**Figure 5:**
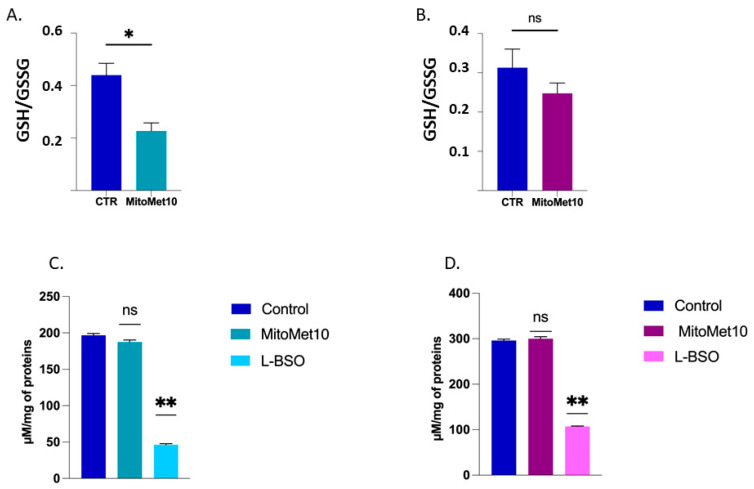
Impact of MM10 on glutathione levels in prostate cancer cells. (**A**) Impact on GSH/GSSG ratio of PC-3 cells after 24 h exposure to MM10 (1 µM). (**B**) Impact on GSH/GSSG ratio of DU-145 cells after 24 h exposure to MM10 (1 µM). (**C**) Impact on GSH_tot_ level of PC3 cells after 24 h exposure to MM10 (1 µM) or L-BSO (25 µM). (**D**) Impact on GSH_tot_ level of DU-145 cells after 24 h exposure to mito-metformin10 (1 µM) or L-BSO (25 µM). L-BSO is an inhibitor of glutathione biosynthesis used as positive control. Bars represent means ± SEM (µM/mg of proteins), (*) *p* < 0.05, (**) *p* < 0.01, (ns) non-significant, Student’s *t* test, N = 3.

The difference observed in superoxide production between the two cell lines prompted us to measure the intracellular levels of glutathione. Interestingly, DU-145 cells presented a higher concentration of total glutathione, GSH_tot_ (glutathione in its reduced and oxidized forms) than PC-3 cells (Figure 4). We also investigated the changes in GSH/GSSG ratio (reduced glutathione on oxidized glutathione ratio) when cells were exposed for 24 h to 1 µM of MM10 to assess their response to oxidative stress. It revealed that the GSH/GSSG ratio was significantly decreased in PC-3 (Figure 5A) cells but not in DU-145 cells (Figure 5B) after MM10 exposure. We also observed that MM10 did not change the levels of GSH_tot_ in PC-3 (Figure 5C) and DU-145 (Figure 5D), contrarily to L-Buthionine-sulfoximine (L-BSO), an inhibitor of glutathione biosynthesis used as positive control.

### 2.4. MM10 Alleviates Tumor Hypoxia in the PC-3 Cancer Model

**Figure 6:**
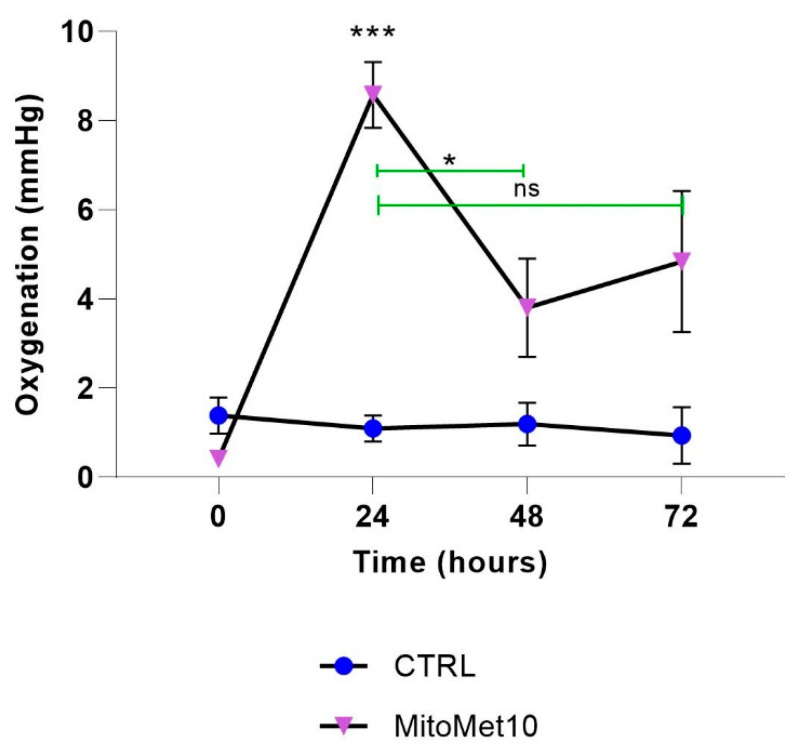
Effect of daily treatment with MM10 (2 mg/kg for 4 days) on tumor oxygenation as measured by EPR oximetry. Data are shown as means ± SEM, (*) *p* < 0.05, (***) *p* < 0.001, Two-way ANOVA, N = 4.

As we observed in vitro a significant decrease in OCR, we hypothesized that MM10 could lead to an increase in tumor oxygenation in vivo, as it was observed previously for other compounds [23,35,36,37]. For the purpose, we measured the oxygenation in PC-3 tumors by in vivo EPR oximetry. We found out that PC-3 tumors were highly hypoxic as the initial pO_2_ before treatment was lower than 2 mmHg (Figure 6). When mice were treated daily with MM10 (IP injection, 2 mg/kg), a sharp increase in tumor oxygenation was observed 24 h after treatment initiation and the tumor oxygenation remained higher than basal pO_2_ up to 72 h (Figure 6). 

### 2.5. MM10 as a Potential Radiosentizer? 

**Figure 7:**
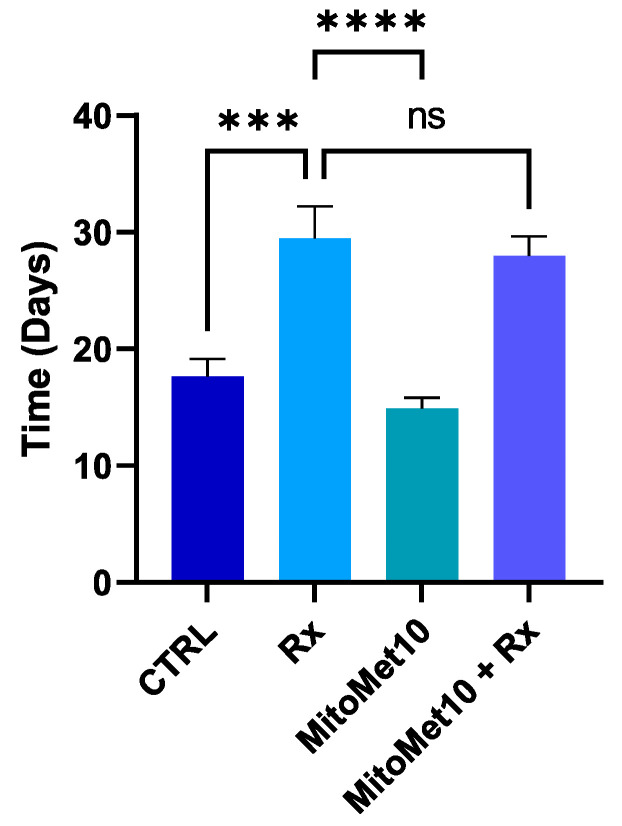
Effect of daily treatment with mito-metformin10 (2 mg/kg) on PC-3 tumor growth delay (in days) after irradiation 6 Gy dose delivered at time of maximal tumor reoxygenation. Data are shown as means ± SEM of tumor growth delay to achieve a 1200 mm^3^ tumor volume. (***) *p* < 0.001, (****) *p* < 0.0001, (ns) non-significant, Two-way ANOVA N = 9/group.

As hypoxia is a major factor of resistance to radiotherapy, we evaluated if MM10 could improve response to radiotherapy when irradiation was administered 1 day after the initiation of MM10 treatment at the time of maximal reoxygenation. Despite the promising results obtained in vitro and in vivo showing the inhibition of mitochondrial respiration and alleviation of tumor hypoxia, the association of MM10 together with irradiation had no additive effect on radiotherapy efficacy in comparison with irradiation treatment alone (Figure 7).

## 3. Discussion

The main result of the present study is that MM10 induced a mitochondrial dysfunction in prostate cancer cells, as it was previously reported for pancreatic adenocarcinomas [12]. However, we can also highlight some similarities and differences between our observation on prostate cancer models and the previous results obtained on pancreatic cancer models.

The inhibition of mitochondrial respiration observed was in the same range of concentration (1 µM) after 24 h exposure in prostate cancer models (Figure 2) and in the PDAC model [12]. In pancreatic adenocarcinoma cells, this interference with the ETC had important consequences in terms of superoxide production, as revealed by fluorescent assays coupled with HPLC [12]. Interestingly, in our EPR study, we observed that the level of mitochondrial superoxide achieved after 24 h exposure to MM10 was strongly dependent on the prostate cancer cell line. The level was significantly increased in PC-3 cells treated by MM10 while it was not modified in DU-145 cells (Figure 3). This observation together with a very low basal level of superoxide (before treatment) in the DU-145 cells suggested that the antioxidant systems may be different in both cell lines. Therefore, we measured the level of glutathione (total GSH and ratio GSH/GSSG). As anticipated, the basal level of GSH was lower in DU-145 cells compared to PC-3 cells (Figure 4). As a consequence, the exposure to MM10 led to a larger decrease in the ratio reduced GSH/oxidized GSSG in the PC-3 model compared to the DU-145 model where it was not significant (Figure 5). Of note, the exposure of cells to MM10 did not modify the level of total GSH, excluding an interference of MM10 with the biosynthesis of glutathione. Altogether, such evidence suggests that the response of cancer cells to the potential oxidative stress induced by MM10 is strongly dependent on their antioxidant capacity. Without excluding other antioxidant systems, such as thioredoxins, peroxyredoxins, glutaredoxins, or superoxide dismutase, as illustrative examples, it suggests that the level of glutathione likely contributes to the modulation of the response to MM10 exposure. Of note, despite the significant increase in superoxide level in PC-3 tumor cells and the alteration of glutathione levels, MM10 treatment did not modify the tumor growth when used alone or in combination with irradiation in this tumor model, contrary to previous observations made in pancreatic adenocarcinomas [12].

Considering the alteration in tumor cell respiration, we anticipated that MM10 treatment could change the concentration of oxygen in tumor models. EPR oximetry experiments revealed a significant increase in tumor oxygenation after daily administration of MM10 (Figure 7). The pO_2_ dramatically increased one day after treatment initiation. The slight decrease observed after the initial peak is likely due to the decrease in perfusion due to the persistent growth of the tumor. The identification of the time window of reoxygenation is essential for the optimal guidance of treatments targeting tumor hypoxia and its consequences [38]. It may help to identify potential treatments, but also give rationale for an appropriate scheduling of irradiation regimen [38,39]. It is well established that the Oxygen Enhancement Ratio (i.e., the ratio of radiation dose to observe a same biological effect) dramatically varies between 1 and 10 mmHg [38,40,41]. As the pO_2_ level increased from 0–1 mmHg to 8–9 mmHg, we designed an experiment with irradiation at the time of maximal reoxygenation. For most experiments done so far using this design, we got an improvement of response using the combination of treatments (reviewed in [19]). However, here, the association of MM10 together with irradiation had no additive effect in terms of tumor growth delay compared to irradiation alone (Figure 7). The elucidation of the reasons for the absence of radiosensitization observed in this tumor model will require further investigation. One limitation of our EPR oximetry study is that it was based on EPR spectroscopy and not EPR imaging. Previous studies have established that, using the present protocol, the charcoal interrogates the oxygenation in a volume of approximately 10 mm^3^ inside the tumor [30]. We cannot exclude that a few hypoxic areas could still be present after the MM10 treatment, remaining resistant to irradiation and be at the origin of the recurrence. In the future, it would be interesting to evaluate this possible heterogeneity of response using EPR imaging [42,43,44,45,46]. We should also keep in mind that oxygen, while being very important, is not the sole factor affecting the response to irradiation. While speculative at this stage, it could result from complex mechanisms, such as the activation of DNA repair mechanisms or cell cycle adaptations in response to radiation. Another lesson from the in vivo experiment is that MM10 (2 mg/kg, used as a single modality without irradiation) did not affect the tumor growth in the prostate cancer model. In contrast, a significant decrease in tumor size was obtained in a pancreatic model [12]. Of note, the administration of MM10 was interrupted after the irradiation in our study while it was prolonged over a longer period in the pancreatic model [12].

Finally, as this paper is submitted for the special issue of *Molecules* on “Application of EPR Spectroscopy in Biophysics and Biochemistry”, we believe that the present study well illustrates the potential role of EPR in trying to identify new pharmacological treatments that may help in fighting cancer. While the final results obtained here are rather disappointing in terms of the potentiation of radiation sensitivity, there are many other examples for which EPR has been instrumental in characterizing the effect of potential treatments [47,48,49,50,51].

## 4. Materials and Methods

### 4.1. Reagents

MM10 was synthetized as described previously [12]. L-Buthionine-sulfoximine (L-BSO) (CAS number 83730-53-4) was from Sigma-Aldrich (Hoeilaert, Belgium). ^15^N-PDT (4-oxo-2,2,6,6-tetramethylpiperidine-d_16_-^15^N-1-oxyl) (CAS 80404-14-4) originated from CDN Isotopes. Mito-TEMPO-H (1-hydroxy-4-[2-triphenylphosphosphonio)-acetamido]-2,2,6,6-tetramethylpiperidine) was from Enzo Lifescience (Brussels, Belgium). Superoxide dismutase conjugated with polyethylene glycol (PEGSOD2), dimethyl sulfoxide (DMSO), diethylenetriaminepentaacetic acid (DTPA) and dextran from leuconostoc mesenteroides (average MW 60,000–76,000) were purchased from Sigma-Aldrich (Hoeilaert, Belgium).

### 4.2. Cell Lines and Culture

The PC-3 and DU-145 cell lines were purchased from the American Type Culture Collection (ATCC) (Manassas, VA, USA) and maintained at 37 °C in a humified atmosphere with 5% CO_2_. PC-3 and DU-145 cells were cultured in Ham’s F12-K (Kaighn’s) and MEM-α without nucleosides media, respectively, with 10% heat-inactivated fetal bovine serum (Thermo Fisher Scientific, Merelbeke, Belgium).

### 4.3. OCR Measurements by EPR Spectroscopy

OCR measurements by EPR spectroscopy were done by using oxygen sensing probe, ^15^N-PDT (4-oxo-2,2,6,6-tetramethylpiperidine-d_16_-^15^N-1-oxyl), to measure variations of oxygen levels in samples, and subsequently cells’ OCR in a sealed capillary [52]. A Bruker EMX-Plus spectrometer (Rheinstetten, Germany) operating in X-band (9.85 GHz) and equipped with a PremiumX ultra low noise microwave bridge and a SHQ high sensitivity resonator was used and the EPR cavity was heated at 310 K with continuous nitrogen flow during all experiments. Into a hematocrit capillary is put a solution containing 60 µL of previously harvested cells (stock solution of 5 × 10^6^ cells/mL of the appropriate culture medium), 40 µL of a 20% dextran solution and 4 µL of ^15^N-PDT at 2 mM (final concentration: 77 µM). The capillary was sealed with gum. EPR parameters set in Bruker Xenon Spin fit program were: microwave power, 2.518 mW; modulation amplitude, 0.005 mT; modulation frequency, 100 kHz; center field, 335 mT; sweep time, 15 s; sweep width, 1.5 mT. An automated “2D-field-Delay” measurement was launched 3 min after probe mixing, counting 15 points with a time delay of 60 s. Data were analyzed by switching to processing mode and using “peak picking” on selected regions of the ^15^N-PDT-peaks. The final file was saved as an ASCII file to extract linewidth data at each point. ^15^N-PDT linewidth was correlated with the % of oxygen with a calibration curve (linewidth under oxygen-free condition: 0.180 mT; sensitivity to oxygen: 0.692 µT/mmHg). OCR corresponded to the slope of evolution of oxygen level as a function of time.

### 4.4. Mitochondrial Superoxide Assessment by EPR Spectroscopy 

To assess the mitochondrial superoxide by EPR, Mito-TEMPO-H, a cyclic hydroxylamine which can detect superoxide in complex biological samples using PEG-SOD as control [26,27,28]. We used a Bruker EMX-Plus spectrometer operating in X-band (9.85 GHz) and equipped with a PremiumX ultra low noise microwave bridge and a SHQ high sensitivity resonator. The EPR cavity was kept at 310 K with continuous air flow during all experiments. We prepared a mixture containing 37 µL of cell suspension previously harvested (stock solution of 1.5 × 10^7^ cells/mL of the appropriate culture medium), 0.5 µL of DTPA (100 mM), 7.5 µL of Mito-TEMPO-H (1 mM) (the solution was flushed with argon before and during pipetting to avoid the probe oxidation). PBS ((1×)-pH 7.4) was added up to a total volume of 50 µL. To isolate the contribution of superoxide to the oxidation of the hydroxylamine into the nitroxide, other measurements were made using the same conditions but adding 2.5 µL of PEG-SOD2 (4000 U/mL) prior Mito-TEMPO-H and letting the mixture incubate 15 min. The final mixture was transferred using a needle in a 12 cm long PTFA tube (inside diameter 0.025 in, wall thickness 0.002 in). The tube was folded 6 times and inserted into an open quartz tube. The EPR parameters set in Bruker Xenon Spin fit program were: microwave power, 20 mW; modulation frequency, 100 kHz; modulation amplitude, 0.1 mT; center field, 336.5 mT; sweep width, 1.5 mT; sweep time, 30.48 s. Measurements were started 3 min after probe mixing with the cells and repeated over time. Data were analyzed by performing a double integration (DI) on selected regions of peaks. Superoxide contribution was measured by subtracting mean PEGSOD2 DI to mean control DI. Full details of the procedure have been published elsewhere [53].

### 4.5. Intracellular Reduced and Oxidized Glutathione Quantification

To measure the glutathione levels, a colorimetric detection kit (catalog num. EIAGSHC) was used (Invitrogen, Thermo Fisher Scientific, Merelbeke, Belgium). PC-3 and DU-145 were seeded 24 h prior treatment at a density of 5 × 10^5^ cells in a 100 mm^2^ petri dish with the appropriate medium. Cells were treated either with DMSO 1 µM (negative control), MM10 1 µM or L-BSO 25 µM (positive control) for 24 h. The levels of total glutathione (GSH_tot_) and oxidized glutathione (GSSG) were assessed following the manufacturer instructions. The levels of reduced glutathione (GSH) were deduced from the data collected for GSH_tot_ and GSSG. For each condition, protein quantification was performed using the BCA Protein Assay Kit (Pierce^TM^, Thermo Fisher). The spectrophotometer used for detection was a SpectraMax M2e plate reader (Molecular Devices). 

### 4.6. Tumor Models In Vivo and Treatments

All experiments involving animals were performed in accordance with the Belgian law concerning the protection and welfare of the animals and were approved by the UCLouvain ethics committee (Agreement reference: 2018/UCL/MD/021).

Male 6–8-week-old NMRI nude mice (Charles Rivers Laboratories, Beerse, Belgium) were housed under standardized conditions of light and temperature (12-hour daylight cycle, 22 ± 2 °C) before and during the experiments. They had ad libitum access to chow pellets and water. After 1 week of acclimatization, 10^6^ PC-3 cells (100 µL cell suspension in HBSS + 100 µL Matrigel (Corning, Glendale, AZ, USA) were inoculated intramuscularly (IM) in the right leg. Tumor size was monitored three times per week using an electronic caliper, and two distances were measured, X and Y (X < Y). Tumor shape was assumed to be ellipsoidal; hence, the volume was considered π/6 × X^2^ × Y^2^. Mice were then randomly allocated to groups when xenograft exceeded a tumor volume of >350 mm³ (3–4 weeks following tumor inoculation) and experiments performed.

### 4.7. In Vivo EPR Oximetry

**Figure 8:**
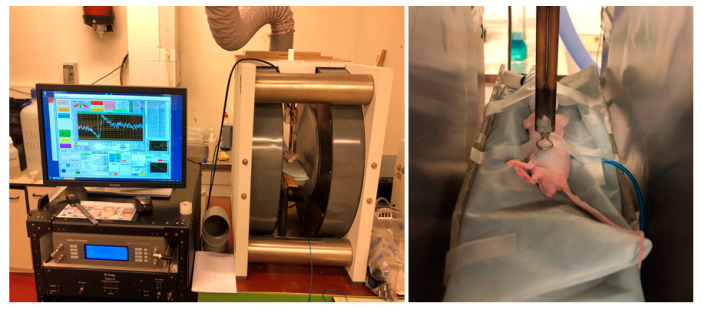
Setup for EPR oximetry measurements. (**Left**) 1 GHz EPR spectrometer. (**Right**) focus on the surface coil put at the surface of a tumor implanted in the leg of an anesthetized mouse.

The application of EPR in vivo requires the use of low-frequency EPR spectrometers. Spectrometers operating at 1 GHz (Figure 8) allow the measurement in tissues with 1 cm depth penetration. To measure the partial pressure of oxygen (pO_2_) in vivo, paramagnetic particles of charcoals were inserted in the tissue of interest one day before the first pO_2_ measurement [30]. Inhalation of isoflurane mixed with air (21% of oxygen) was used to anesthetize animals with a continuous flow (2 L/h). Induction was performed with 3% of isoflurane and anesthesia was maintained with 1.5% for at least 15 min before measurement. It was previously demonstrated that this anesthesia regimen does not disturb hemodynamics in rodents [54]. The body temperature was kept at 37 °C by a warm water blanket. Tumor oxygenation was measured by using a charcoal CX 0670-1; EM Sciences, Gibbstown, NJ, USA) as oxygen sensor [55] to dynamically evaluate changes in tumor oxygenation during mito-metformin treatment. EPR spectra were recorded with an EPR spectrometer (electromagnet from Magnettech, Berlin, Germany; electronic console from Clin-EPR, Lyme, NH, USA) with a low frequency microwave bridge operating at 1.1 GHz. The EPR spectrum was recorded noninvasively using a loop surface coil [56] placed over the tumor of an anesthetized mouse (Figure 8). Hence, 50 µL of a suspension of charcoal (100 mg/mL, particle size of 1–25 µm) was injected into the center of the tumor. Four mice were allocated in each group (treatment or control). Mice were anesthetized and baseline values were recorded to obtain the oxygen status of tumors before treatment. Levels of oxygen were obtained thanks to a calibration curve to transform EPR linewidths into pO_2_ values [55]. The effect of MM10 (2 mg/kg) on tumor oxygenation was measured daily, 5 min after the mito-metformin IP injection. 

### 4.8. Tumor Growth-Delay

Briefly, 36 male NMRI PC3-tumor bearing mice were randomly allocated into 4 groups (control, RX (irradiation of 6 Gy, MM10, MM10 + Rx) of 9 mice with the same mean tumor volume (421 ± 53 mm^3^, mean ± standard deviation). A single irradiation dose of 6 Gy was delivered at the time of maximal reoxygenation (24 h after 2mg/kg IP injection). Tumor volume was measured 3 times a week using a caliper to assess tumor growth-delay.

### 4.9. Statistics

Data are represented as means ± SEM. All experiments were performed in triplicates or more. Statistical tests were performed using *GraphPad Prism* software version 9.1 (San Diego, CA, USA) and are noted under each figure.

## Data Availability

All results are reported in the manuscript.
